# Piezoelectric extraction of ECG signal

**DOI:** 10.1038/srep37093

**Published:** 2016-11-17

**Authors:** Mahmoud Al Ahmad

**Affiliations:** 1 Department of Electrical Engineering, College of Engineering, UAE University, P.O. Box 15551, Al Ain, UAE

## Abstract

The monitoring and early detection of abnormalities or variations in the cardiac cycle functionality are very critical practices and have significant impact on the prevention of heart diseases and their associated complications. Currently, in the field of biomedical engineering, there is a growing need for devices capable of measuring and monitoring a wide range of cardiac cycle parameters continuously, effectively and on a real-time basis using easily accessible and reusable probes. In this paper, the revolutionary generation and extraction of the corresponding ECG signal using a piezoelectric transducer as alternative for the ECG will be discussed. The piezoelectric transducer pick up the vibrations from the heart beats and convert them into electrical output signals. To this end, piezoelectric and signal processing techniques were employed to extract the ECG corresponding signal from the piezoelectric output voltage signal. The measured electrode based and the extracted piezoelectric based ECG traces are well corroborated. Their peaks amplitudes and locations are well aligned with each other.

Monitoring of the heart’s mechanical and electrical dynamics is essential to fully characterize and understand the heart functions and variations^1^. Attention so far has been given to the assessment of the biophysical properties of the heart’s components through the use of conventional measurement approaches such as ECG. ECG is usually used to obtain measurements for different heart parameters^2^. It is normally used in a procedure which facilitates the recording of the electrical activity of the heart muscle during a specific time interval. In this procedure, multiple probes are placed at defined locations on a bare chest. Those probes generate electrical current as a result of measuring the electrical activity stemming from each heartbeat at the chest surface^3^.

Various applications have been developed to measure vital signs by means of piezoelectric material^4^,^5^,^6^,^7^,^8^. A piezoelectric based system has been developed to monitor respiration rate^7^, heart rate^4^,^5^,^6^, and seismocardiography^8^. This paper presents a novel technique for the reconstruction of the ECG corresponding signal from a measured piezoelectric output voltage signal. The motivation behind this work rests on the following fact; both piezoelectric sensor and ECG produce voltage output signals for the parameter they are measuring. Thus, for the purpose of this work, it was found convenient correlating the two signals using signal processing techniques such as convolution theorem^9^ and Fourier transform^10^.

## Piezoelectric ECG extraction approach

**Figure 1:**
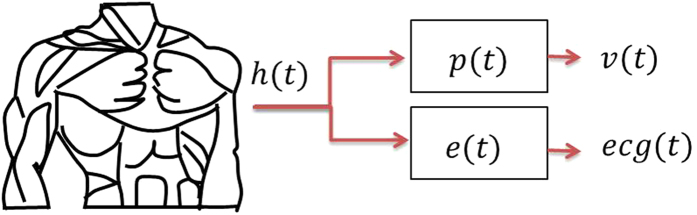
Schematic overview of the presented approach.

Figure 1 represents a schematic overview of the proposed approach. In principle, the heart mechanical activities generate electrical potentials that can be detected on the surface of the body using electrodes attached to the chest. At the same time, these activities induce periodic vibrations inside the chest which can be sensed and collected also at the chest surface using piezoelectric sensors. Both ECG electrode system and piezoelectric transducer responses are electrical in nature. Hence, the system of Fig. 1 could be modeled as a single input multiple outputs, where: *h(t*) is the system input and *v(t*), *ecg(t*), are the system piezoelectric and ECG outputs, respectively.

To establish a direct link between the ECG trace and the piezoelectric signal; the piezoelectric output voltage *v(t*) can be presented as a convolution process of *h(t*) and the impulse response of the piezoelectric transducer, *p(t*). On the other hand, the ECG output voltage *ecg(t*) can be presented as a convolution process of *h(t*) and the impulse response of the ECG electrode system, *e(t*). Mathematically, these relationships could be represented as follow:


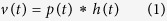



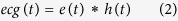


where * denotes convolution process^9^.

The direct connection between *v(t*) and *ecg(t*) can be then be constructed using the well-known Fourier transformation^10^. Equation (3) and (4) represent the corresponding Fourier transformation of equation (1) and (2), respectively:


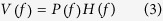



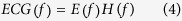


where *V(f*), *ECG(f*) and *H(f*) are the corresponding Fourier signal transform of *v(t*), ecg(*t*) and *h(t*), respectively. *P(f*) represents the frequency response of the piezoelectric transducer and *E(f*) represents the frequency response of the ECG system. Therefore, rearranging (3) for *H(f*) yields:


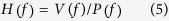


Substituting (5) in (4), produces:


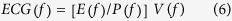


This could be simplified as follow:


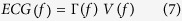


where Γ(*f*) = *E(f*)/(*P(f*). Equation (7) represents the direct link between ECG and PZE signals and could be used to transform the measured piezoelectric voltage signal to electrocardiography trace provided that the transfer function Γ is known. To determine Γ(*f*); both piezoelectric and electrocardiography time domain traces are simultaneously measured for only one time. Then, the frequency domain signal of *ECG(f*) is divided by the frequency domain signal (*f*). This is how Γ(*f*) is computed. Furthermore, this computation is performed once and then to construct the corresponding *ECG* time domain trace at any time later from an instant piezoelectric voltage, the following relationship is used:


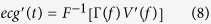


where F^−1^ is the inverse Fourier transformation, *V*′(*f*) is the corresponding frequency domain of the instant piezoelectric voltage. Equation (8) demonstrate the possibility of using piezoelectric transducer to replace a multi wire ECG system that allows the piezoelectric transducer time domain voltage signal to be mapped to an equivalent ECG time domain trace.

### Piezoelectric transducer and experimental setup

In this section, a brief description about the used piezoelectric transducer will be given followed by the details of the experimental setup.

### Piezoelectric transducer

The main motivation behind the use of piezoelectric transducer is its capability to map conformally the acting mechanical load to electrical voltage. Additionally, due to the nature of the involved piezoelectric materials and the fact that the heart mechanical contractions and expansions actions are cyclical in nature, the piezoelectric transducer produced output voltage that reflects this cardiac cycle. When the piezoelectric sheet which is placed on the anterior chest surface is subject to a mechanical load produced by the heart muscles contractions and expansions, the strain induced in the piezoelectric material generates a voltage^11^. The relation between the stress acting on the piezoelectric transducers, represented by *σ*_*H*_(*t*), and output voltage signal, *v(t*), is given by (9), where n is the piezoelectric turn ratio representing the mechanical to electrical conversion process in the transducer^11^:


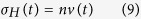


where


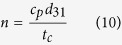


where: *c*_*p*_, *t*_*c*_ and *d*_31_ are the elasticity coefficient, thickness and piezoelectric voltage constant, respectively. Thus the acting stress on the piezoelectric transducer is computed using equation (9) by knowing the dimensions and involved material parameters of the transducer along with its corresponding output voltage and its corresponding first time domain derivative.

**Figure 2:**
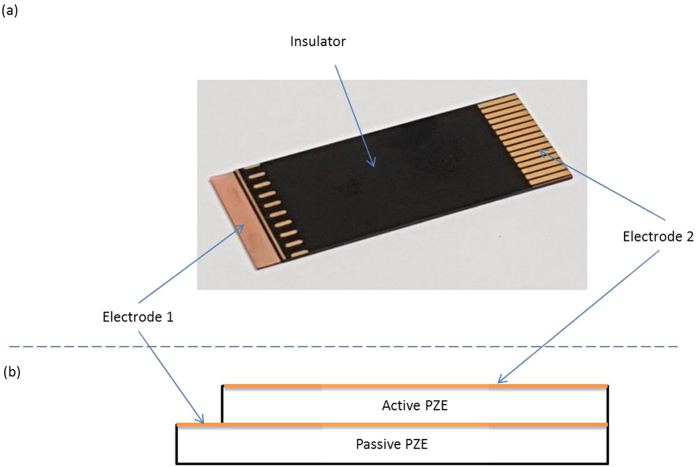
Piezoelectric transducer: (**a**) photo of the used transducer and (**b**) its cross section.

Figure 2(a,b) shows the design of the piezoelectric bimorph transducer employed in this work and its cross section. The material has a d_31_ of 315 pm/V. The transducer has a size of 46 mm in length, 20 mm in width, and 0.26 mm in thickness with composition of lead-zirconate-titanate (PZT) and has a bimorph structure. Specifically, the transducer is a bimorph piezoelectric beam custom made by Johnson Matthey in their M1100 ceramic^12^.

The piezoelectric is composed of two piezoelectric layers. As shown in Fig. 2(b), the top layer is active one that is used to convert the heart activity to electrical output signal. This output is taken between electrode 1 and 2. The bottom layer is passive one and is needed for isolation to avoid an electrode contact with the chest surface.

### Experimental setup

**Figure 3:**
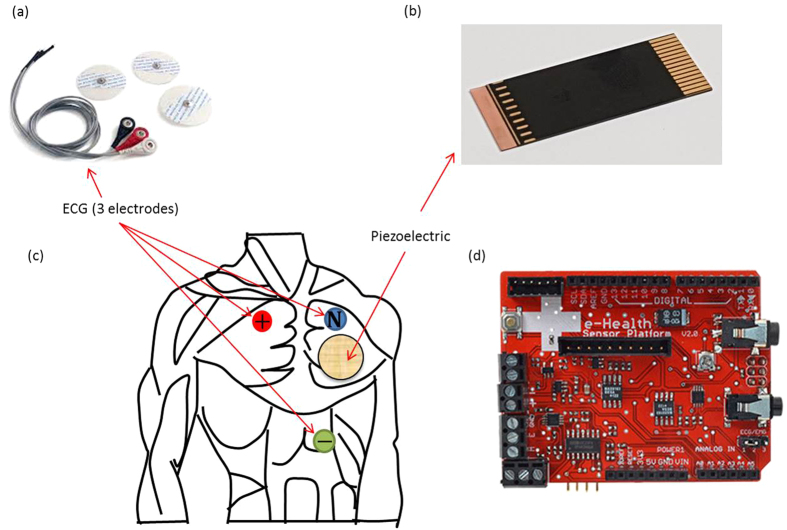
Devices and experimental setup: (**a**) three electrode ECG system^13^, (**b**) a piezoelectric sensor^12^, (**c**) measurements setup illustration and (**d**) eHealth platform shield^13^.

The data acquisition system itself is depicted in Fig. 3. The e-health sensor platform kit^13^ has been used in this work. The main reason for choosing this sensor platform kit is because of built in conditional circuit which does amplification and provides DC offset that is needed for the microcontroller. It can support multiple sensor readings on the same time, which makes it possible to record both PZE and ECG sensor reading simultaneously.

**Figure 4:**
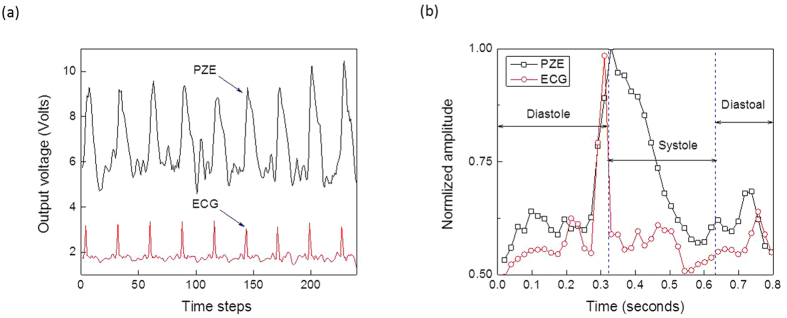
Measured output signals of ECG and piezoelectric sensors: (**a**) Piezoelectric (PZE) and ECG output signals and (**b**) normalized signals for single cardiac cycle.

The connections of the ECG electrodes along with the piezoelectric sensor placement at the surface of the chest are illustrated in the measurements setup depicted in Fig. 3(c). The eHealth Sensor Shield V2.0 platform should be placed on top of a microcontroller (Arduino Uno^14^). The eHealth shield analog input pins are connected directly to Arduino analog input pins. The analog input pins were used to acquire the piezoelectric signal. Using CoolTerm^15^ serial port terminal application the two sensors data were logged and saved in a data file. The superimposed plotting of the simultaneous readings from both sensors (ECG and piezoelectric) is illustrated in Fig. 4(a). It must be noted that acquiring the piezoelectric sensor signal require the subject (human) undertaking the test to hold his breath while conducting the experiment. This is due to the high sensitivity of the piezoelectric sensor. It can detect human breathing pattern while acquiring the ECG signals which apparently can interfere with the required heart activity signals. It is worth to add that in the ECG conventional method and for continuous monitoring; one may be asked to hold his/her breath for short periods during the procedure (to stop the movement of chest wall interfering with the signal)^16^.

## Results and Discussion

In this section, the transfer function construction will be explained briefly and the ECG trace extraction results will be discussed.

### Transfer function construction

**Figure 5:**
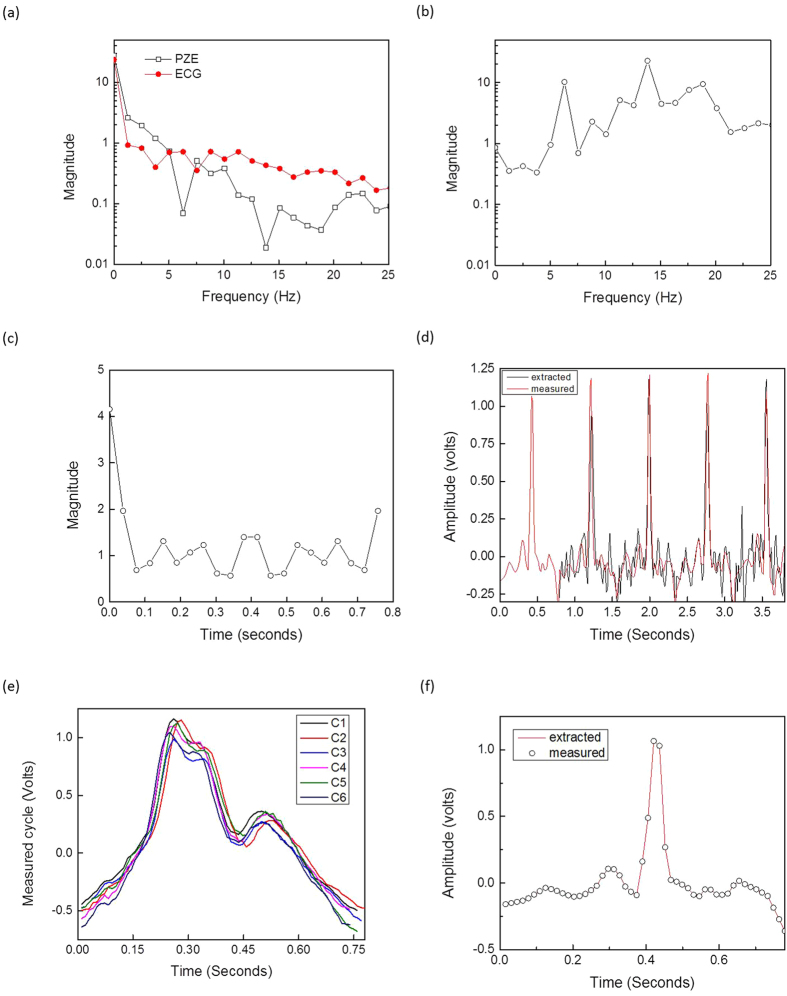
Signal processing: (**a**) frequency domain of ECG and piezoelectric output voltages. (**b**) Constructed frequency response of Γ(*f*). (**c**) Time domain impulse response of the transfer function. (**d**) Comparison between extracted and measured ECG signal. (**e**) Superimposed cycles with the reference one (C1) and (**f**) ECG extracted and measured cycle after adjustment.

As shown in Fig. 4(a), the PZE and ECG signal peaks are aligned well with each other. Therefore, based on these reported facts, the piezoelectric response can be directly mapped to the cardiac cycle physiology. Figure 4(b) shows the normalized measured ECG and piezoelectric output voltages along with their corresponding cardiac polarization, depolarization phases. It also shows that both piezoelectric and ECG signals are of a periodic nature with a time interval of 0.8 second. Based on the data reported in Fig. 4, the magnitude of the Fourier transform versus frequency for both the piezoelectric and ECG output voltage signals have been extracted and are plotted in Fig. 5(a). Furthermore, Γ(*f*) transfer function versus frequency has been computed and it is depicted in Fig. 5(b). Specifically, Fig. 5(b) represents the typical frequency response of a single complete normal cardiac cycle connection between the piezoelectric and the ECG signal for a specific person. This function could vary from person to person depending on individual’s health condition, age, gender, weight … etc. Then, the corresponding time-domain impulse response is constructed using the inverse Fourier transform theory. The real time domain response is plotted in Fig. 5(c). This transfer function (Γ(*f*)) is performed one-time and stored to be used as a reference for instantaneous piezoelectric ECG extraction signal. It is important to note that the construction of the transfer function must be carried out when the subject is well and not suffering from any heart problems in order to be able to discriminate later if there is some divergence.

### ECG trace extraction

The measured and extracted ECG traces are superimposed as shown in Fig. 5(d). The extracted ECG shows a good agreement with the measured one. The peaks amplitudes and locations are well aligned in both signals. The extracted first cycle matches 100% because it was the same cycle used to find the transfer function. The other cycles show a little deviation. Figure 5(e) depicts six piezoelectric cycles that are not aligned with the reference cycle (C1). To solve this issue, it is important to tune the instantaneous measured cycle in the time domain until it achieves the maximum matching with the reference cycle. This maximum matching search can be conducted using the correlation principle^17^. The instantaneous measured cycle is shifted in time domain and correlated with the reference cycle. Figure 5(f) depicts the extracted signal superimposed with the corresponding measured one after the adjustment. The two signals are now well matched and their similarity is close to 100%.

The above experiment results demonstrate the possibility of extracting ECG corresponding signal using piezoelectric transducers. The proposed approach will pave the way towards a new generation of contactless, affordable and reusable probing tools for ECG signal extraction which in turn will facilitate wireless detection and prediction of any heart failure and abnormalities. The piezoelectric sensor has many advantages. When compared with the standard ECG technique, piezoelectric sensor needs shorter processing time and lower processing power. Furthermore, the ECG electrodes cannot be reused and must be disposed directly after use. Reusing them will render inaccurate results while the piezoelectric sensor is reusable and non-disposable. Moreover, the minimum number of probes required to acquire an adequate ECG signal is three^18^, which makes the procedure inconvenient for home-based patients without trained caregiver support. On the other hand, one piezoelectric sensor is sufficient to do almost a comparable job.

## Conclusions

In summary, a systematic procedure for the extraction of ECG signal using piezoelectric sensor has been proposed. The piezoelectric signal is conformally mapped to the heart physiological activity. As shown, the heart mechanical activities can be collected in terms of electrical charges at the chest surface by either piezoelectric transducer or ECG based electrode setups. The demonstrated piezoelectric ECG trace extraction approach can replace the conventional ECG electrode based setup with an efficient, cheap and easy to use solution.

## Method

- The authors confirm that all methods were carried out in accordance with relevant guidelines and regulations.

- The authors confirm that all experimental protocols were approved by *Al Ain Medical District Human Research Ethics Committee* – Protocol N0. 14/67- cardiac and inspiratory piezoelectric study.

- The authors are confirming that informed consent was obtained from all subjects.

## Additional Information

**How to cite this article**: Ahmad, M. A. Piezoelectric extraction of ECG signal. *Sci. Rep.* **6**, 37093; doi: 10.1038/srep37093 (2016).

**Publisher’s note:** Springer Nature remains neutral with regard to jurisdictional claims in published maps and institutional affiliations.
